# Associations between optimism, tobacco smoking and substance abuse among Iranian high school students

**DOI:** 10.15171/hpp.2019.38

**Published:** 2019-10-24

**Authors:** Soudabeh Marin, Esmaeil Heshmatian, Haidar Nadrian, Ali Fakhari, Asghar Mohammadpoorasl

**Affiliations:** ^1^Department of Statistics and Epidemiology, Tabriz University of Medical Sciences, Tabriz, Iran; ^2^Department of Health Education, Tabriz University of Medical Sciences, Tabriz, Iran; ^3^Social Determinants of Health Research Center, Tabriz University of Medical Sciences, Tabriz, Iran; ^4^Research Center of Psychiatry and Behavioral Sciences, Tabriz University of Medical Sciences, Tabriz, Iran; ^5^Tabriz Health Services Management Research Center, Tabriz University of Medical Sciences, Tabriz, Iran

**Keywords:** Substance abuse, Optimism, Adolescent, Tobacco smoking

## Abstract

**
Background:** Optimism is known to be associated with many health behaviors. However, the associations between optimism, tobacco smoking and substance abuse in adolescents are not well documented. This study aimed to address this research gap in a large school-based population.

**Methods:** Participants (N = 1104) were selected based on multi-stage cluster sampling method. Cigarette and hookah smoking behaviors, illicit drug use, optimism, and relevant covariates were measured using a validated questionnaire. Data were analyzed using ordinal logistic regression.

**Results:** After adjustment, higher optimism score was a protective factor against being situated in advanced stages of cigarette smoking (odds ratio [OR] = 0.88, 95% CI: 0.84-0.91), hookah smoking (OR = 0.91, 95% CI: 0.88-0.94), and illicit drugs usage (OR = 0.90, 95% CI: 0.85-0.95). Moreover, the results showed that negative-stability and negative-globality domains of optimism were significantly higher among advanced-stage smokers and illicit drug users.

**Conclusion:** Optimism was found to be a protective factor against tobacco smoking and substance abuse; whereas pessimism (negative-stability and negative-globality) was found to be a determinant factor. Further research is needed to investigate the effects of optimism on the transition in cigarette and hookah smoking stages.

## Introduction

Tobacco smoking, as a preventable cause of death, disability and economic loss, is a global public health concern.^1^ It is well-known that the initiation of tobacco smoking in adolescence increases the likelihood of regular smoking in adulthood.^2^ According to previous studies, the individuals who had smoked during their childhood, were five times more likely to continue smoking in adulthood, and those who started smoking in early adolescence were more likely to become heavy smokers in the future.^3^ Hence, a better understanding on the determinants of tobacco smoking and illicit drug use in adolescents are fundamentally needed for development of further preventive programs. Although several studies have shown psychological, social, and demographic factors related to smoking onset and smoking status,^4-6^ little is known about the association between optimism, tobacco smoking, and substance abuse in adolescents.

A generalized expectation of positive future events is regarded as optimism.^7^ An optimistic view makes it possible for an individual to assess stressful situations with positive thinking and to cope effectively with an adversity.^8^ Optimistic explanatory style was derived from the helplessness model and attributional style, which indicates how individuals explain the cause of positive and negative events.^9,10^

The association between optimism and psychological and physical health have been evident in some studies.^11,12^ For instance, lack of optimism is a risk factor for coronary heart disease.^13^ Results of the Woman’s Health Initiative (WHI) study showed that optimism is related to the incidence of coronary heart disease, depression and postmenopausal mortality.^14,15^ In addition, unhealthy diet, alcohol consumption, smoking and obesity have also been associated with low optimism scores.^16^

This study aimed to examining the relationship between optimistic explanatory style and cigarette smoking, hookah smoking, and illicit drug use among high school students in Sonqor county, Iran.

## Materials and Methods

### Study setting and participants

This cross-sectional study was conducted in Sonqor county (Kermanshah province), Iran, from February 2018 to March 2018. Using STATA software and considering the *P* = 0.054 (prevalence of regular smoking), α = 0.05, and d = 0.02, the required sample size was estimated to be 456. Considering the cluster sampling (design effect =2), missing data (20%), the minimum sample size was estimated as 1095. Participants were selected based on a multi-stage cluster sampling method. At first, half of high school groups (46 groups) in the county were randomly selected after considering the grade, gender, and major of the students. Then, all students in the selected groups (n = 1104) were invited to participate in the study. Five-hundred sixty-four (55.2%) participants were male. Students completed an anonymous, self-administered and validated questionnaire. The items of questionnaire included optimism, status of cigarette and hookah smoking, drug abuse, having smoker friend(s), having smoker member(s) in the family as well as demographic and socio-economic characteristics.

### Measures

Optimism was measured using the Children Attributional Style Questionnaire (CASQ). The CASQ is a 48-item instrument designed to evaluate the causal explanation for positive and negative events. The CASQ is composed of three dimensions: internality (internal-external), stability (stable-unstable) and globality (global-specific). Each dimension is assessed by an equal number of items for positive and negative situations (n = 8). Each item includes a hypothetical situation followed by two statements inquiring why the situation happened. Each internal, stable and global attribution of causality to the situation grants a score of 1 and each external, unstable and specific attribution grants a score of 0. Subscales are scored by summing the values of items in each dimension. Total optimism score is calculated by summing the three dimension scores.^17^ Higher scores represent higher optimism level. The CASQ has demonstrated good criterion-related reliability, test-retest reliability (r = 0.53, *P* < 0.001), and moderate internal consistency (Cronbach’s alpha = 0.58).^18^

A valid algorithm was used to determine the participant’s cigarette status.^19^ According to this algorithm, participants were classified into 3 categories: never *cigarette*smoker (students who have never tried smoking of cigarette); cigaretteexperimenter (students who had tried or smoked less than 100 cigarettes); and regular *cigarette*smoker (students who have smoked 100 cigarettes or more in their lifetime).

A similar classification was used to assess hookah smoking status. Students were categorized into 3 groups of hookah smoking as following: never hookah smoker (students who have never smoked hookah - even a puff), hookah experimenter (students who have tried hookah smoking or have smoked occasionally) and regular hookah smoker (students who use hookah at least once a month).

The students were classified as illicit drug users if they reported that the use of opium, cannabis, ecstasy, and methamphetamine in their lifetime.

Socioeconomic status (SES) was built using principal component analysis. Father education, mother education, family assets, and family income were variables included in the analysis. Subsequently, students were divided into three different SES levels: high, medium and low.

### Statistical analyses

Data were presented using mean (standard deviation) for the numeric variables and frequency (percent) for categorical variables. A series of χ^2^ tests was used to assess the relations between cigarette smoking status, hookah smoking status, and illicit drug abuse and the qualitative variables. The relationships between optimism and cigarette smoking status, hookah smoking status and illicit drug abuse were assessed using one-way ANOVA and independent samples *t* test. Ordinal logistic regression was used for multivariate analysis. Directed Acyclic Graph (DAG)^20^ was used to determine potential confounders. DAGitty (http://dagitty.net), a web-based software, was used to analyze the DAG.^21^ Potential confounders were tested separately in each of the three models. Stata software version14 (StataCorp, Texas, US) was used for all data analyses.

## Results

The results showed that 764 (76.9%), 156 (15.7%) and 74 (7.4%) students were never cigarette smokers, cigarette experimenters, and regular cigarette smokers, respectively. In terms of hookah smoking, 639 (60.6%) students never smoked hookah, 333 (31.5%) were hookah experimenters, and 83 (7.9) participants were regular hookah smokers (i.e., smoking hookah at least once per month). One hundred and five students (9.9%) the use of some kind of illicit drugs during their lifetime (see Table 1).

Table 2 presents the mean and standard deviation for optimistic explanatory style subscales by cigarette smoking status, hookah smoking status, and illicit drug use. Individuals who had used illicit drugs and those who were at advanced stages of cigarette and hookah smoking consumption had significantly different scores in the negative-stability and negative-globality domains, compared to never tobacco smokers and never illicit drug users.

**Figure 1:**
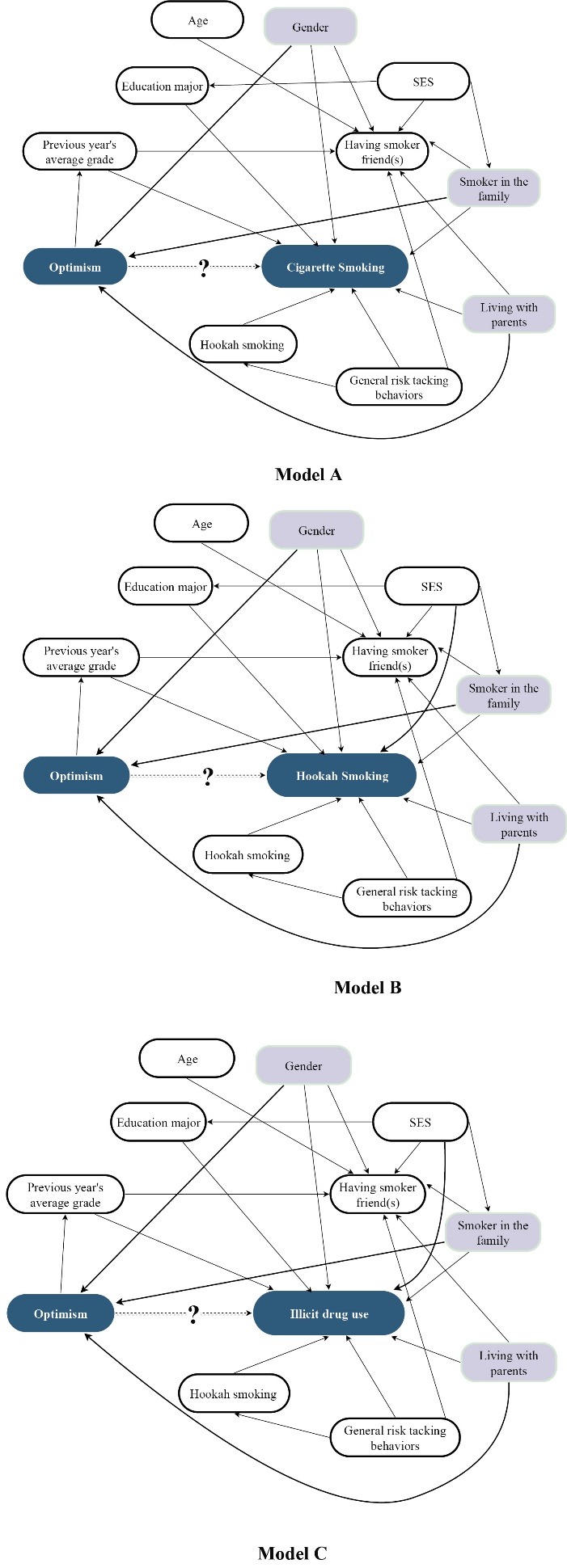
Causal diagram for the association between optimistic explanatory style and cigarette smoking (Model A), hookah smoking (Model B) and illicit drug use (Model C) and other factors. According to the study data, a minimum set of confounders would include gender, a smoker in the family and living with parents.

To investigate the relationship between optimism score and cigarette smoking status, hookah smoking status, and illicit drug use, three ordinal logistic regressions were conducted (Table 3). Based on the DAGitty analysis (see Figure 1), gender, having at least one smoker in the family, and living with parents were entered as confounder variables into the models.

According to the model, higher scores of optimism protect students from being in advanced levels of cigarette smoking (Model 1: OR = 0.88, 95% CI: 0.84-0.91, *P* < 0.001); being in advanced stages of hookah smoking (Model 2: OR = 0.91 95% CI: 0.88-0.94, *P* < 0.001); and using illicit drugs (Model 3: OR = 0.90, 95% CI: 0.85-0.95, *P* < 0.001).

## Discussion

The present study investigated the relationships between optimistic explanatory style and cigarette smoking status, hookah smoking status, and illicit drugs use among high school students in Sonqor county, Iran. Our results showed that never-tobacco smokers had higher levels of optimism compared to the experimenters and regular smokers. Consistent with our study, previous studies have also shown that optimism is associated with being a non-smoker.^16,22,23^ A study in the United States showed that less optimistic individuals were more likely to smoke cigarettes, compared to more optimistic individuals.^24^

Our results stand in contrast with the findings from a study conducted in the United States within which no significant difference was found in optimistic explanatory style between smoker types.^25^ This contrast may be due to multiple reasons. As instances, in contrast with our study, in the study performed in the United States an online survey were performed, participants varied widely in age (from 22 to 45 years old), and the smokers were classified based on the frequency and amount of cigarettes they smoked. Moreover, the researchers did not report the participation rate in their study. It is possible that individuals who regularly smoked or had low levels of optimism were less interested to participate in the study. Tyc et al, investigating predictors of smoking among adolescents, showed also no significant relation between optimism and smoking status.^26^ In contrary to our study, they however used a different optimism theory and assessment instrument, the Youth Life Oriented Test (LOT), to investigate optimism among adolescents. The LOT is an instrument that assesses dispositional optimism,^27^ which refers to generalized outcome expectancies that positive events, rather than negative events, may be happened.^11^

Several studies have shown that unrealistic optimism causes the smokers to underestimate the risk of lung cancer and other health risks and thus they are less likely to plan for quit smoking.^28-30^ While dispositional optimism and optimistic attribution style, that investigated in the present study, are distinct from unrealistic optimism, both constructs have positive effects on morbidity and mortality.^31,32^ Unrealistic optimism occurs when an individual perceives his/her risk for a problem more favorable (i.e., below average), compared to the risk of others for the problem.^33^ In contrast with dispositional optimism and optimistic explanatory style, unrealistic optimism may have unwanted or harmful consequences, including taking more risky behaviors, fewer health precautions, and emotional costs.^34^

According to the results of present study, individuals who were in higher levels of cigarette and hookah smoking and those who have used illicit drugs had higher scores in the negative-stability dimension of optimism (regular smokers >experimenter smokers >never smokers), and lower scores in negative-globality dimension. Stability refers to the perception that an outcome is seen as permanent or temporary. Globality refers to the occurrence of a cause in a broad or narrow range of situations.^9^ Negative-stability and negative-globality are defined as measures of pessimism.^35^ Accordingly, regular- and experimenter smokers and illicit drug users are more pessimistic than never smokers and never drug-users. Therefore, pessimism may be a risk factor for tobacco smoking. Further longitudinal studies are required to investigate the relative risk of pessimism and optimism in the transition between smoking levels.

This cross-sectional study showed that optimism was inversely associated with cigarette smoking status, hookah smoking status and using illicit drugs. Accordingly, the lower the optimism score was, the higher were the stages of cigarette smoking status, hookah smoking status and using illicit drugs.

Although optimism is partially inheritable, it can be shaped and learned through social influences.^36,37^ Thus, planning optimism improvement interventions for adolescents, who are at critical ages of smoking initiations, might provide a useful alternative in smoking and substance abuse reduction and general health promotion. A previous study indicated that the development of optimism may positively impact performance at workplace.^38^ It was also indicated that optimism changes may be negatively associated with self-related health and chronic illnesses over time.^39^ Future intervention studies are needed to investigate the effectiveness of optimism development intervention in onset and transition in smoking status.

### Strengths and limitations

As strengths for our study, large sample size and high response rate could provide generalization for the study. Moreover, an anonymous questionnaire was used to reduce common-method bias. As a limitation of the study, the cross-sectional design of the study may be noted was we could not interpreted the causal relationships between the variables. Future longitudinal studies are required to confirm the findings and investigate causal relationships. Low optimistic perspective, being smoker and being absent at the time of study were effective factors for participation in the study. Hence, selection bias is possible in the present study.

## Conclusion

Optimism was found to be a protective factor against tobacco smoking and substance abuse; whereas pessimism (negative-stability and negative-globality) was found to be a determinant factor. Further research is needed to investigate the effects of optimism on the transition in cigarette and hookah smoking stages.

## Ethical approval

This study was approved by the Ethics Committee of Tabriz University of Medical Sciences (Ethical code: IR.TBZMED.REC.1396.1105) and the Research Committee of the East Azerbaijan Province Education Organization.

## Competing interests

The authors declare that they have no competing interests.

## Funding

This study received financial support from Tabriz Health Services Management Research Center, Tabriz University of Medical Sciences, Tabriz, Iran. This research center had no role in study design, collection, analysis or interpretation of data, or writing the manuscript.

## Authors’ contributions

All authors contributed equally in study conceptualization and design, results interpretation, drafting and revision of the manuscript. EH contributed to acquisition of data. EH, SM and AM contributed to statistical analysis. AM, AF and HN contributed to critical revision.

## Acknowledgements

The authors would like to greatly acknowledge financial support for this study from Tabriz Health Services Management Research Center, Tabriz University of Medical Sciences. The authors also wish to thank all the participants of this study for their valuable cooperation and participation.

**Table 1:** Cigarette- and hookah smoking status and illicit drug use by gender

**Substance**	**Boys** **No. (%)**	**Girls** **No. (%)**	**Total** **No. (%)**	**95% CI**
Cigarette				
Never Smoker	389 (70.9)	375 (84.3)	764 (76.9)	74.1-79.4
Experimenter	99 (18.0)	57 (12.8)	156 (15.7)	13.5-18.0
Regular Smoker	61 (11.1)	13 (2.9)	74 (7.4)	0.6-9.2
Hookah				
Never Smoker	315 (52.2)	324 (71.8)	639 (60.6)	57.5-63.5
Experimenter	215 (35.6)	118 (26.2)	333 (31.5)	28.8-34.4
Regular Smoker	74 (12.3)	9 (2.0)	83 (7.9)	6.4-9.6
Illicit Drugs				
No	527 (86.8)	425 (94.4)	952 (90.1)	88.1-91.7
Yes	80 (13.2)	25 (5.6)	105 (9.9)	8.2-11.8

**Table 2:** Optimism subscale’s score by cigarette smoking status, hookah smoking status and illicit drug use

**Optimism dimensions**	**Cigarette smoking status**			***P***	**Hookah smoking status**			***P***	**Illicit drug use**		***P***
	**Never smoker**	**Experimenter**	**Regular smoker**		**Never smoker**	**Experimenter**	**Regular smoker**		**No**	**Yes**	
	**Mean ± SD**	**Mean ± SD**	**Mean ± SD**		**Mean ± SD**	**Mean ± SD**	**Mean ± SD**		**Mean ± SD**	**Mean ± SD**	
Positive stability	3.87 ± (1.39)	3.35 ± (1.50)	3.40 ± (1.36)	<0.001	3.84 ± (1.39)	3.60 ± (1.46)	3.46 ± (1.35)	0.009	378 ± (1.41)	3.41 ± (1.39)	0.011
Negative stability	2.83 ± (1.49)	3.50 ± (1.39)	4.2 ± (1.56)	<0.001	2.83 ± (1.50)	3.30 ± (1.50)	4.00 ± (1.72)	<0.001	2.96 ± (1.51)	4.12 ± (1.55)	<0.001
Positive globality	4.54 ± (1.45)	4.46 ± (1.38)	4.40 ± (1.53)	0.659	4.57 ± (1.45)	4.35 ± (1.40)	4.50 ± (1.44)	0.052	4.52 ± (1.44)	4.47 ± (1.48)	0.713
Negative globality	2.56 ± (1.37)	3.03 ± (1.40)	3.56 ± (1.61)	<0.001	2.61 ± (1.42)	2.84 ± (1.44)	3.56 ± (1.47)	<0.001	2.68 ± (1.40)	3.37 ± (1.60)	<0.001
Positive internality	4.76 ± (1.31)	4.69 ± (1.31)	4.23 ± (1.47)	0.005	4.76 ± (1.33)	4.73 ± (1.30)	4.23 ± (1.36)	0.003	4.74 ± (1.32)	4.45 ± (1.41)	0.035
Negative internality	3.62 ± (1.50)	3.63 ± (1.32)	3.49 ± (1.37)	0.757	3.57 ± (1.50)	3.69 ± (1.46)	3.78 ± (1.17)	0.331	3.64 ± (1.47)	3.48 ± (1.40)	0.321
Total optimism score	4.16 ± (4.01)	2.34 ± (4.07)	0.77 ± (4.60)	<0.001	4.17 ± (4.02)	2.86 ± (4.15)	0.99 ± (4.37)	<0.001	3.77 ± (4.08)	1.34 ± (4.65)	<0.001

**Table 3:** Ordinal logistic regression analysis of the relationships between “cigarette smoking stages”, “hookah smoking status” and “illicit drug use” with “optimism”

**Optimism score**	**Cigarette smoking stages**		**Hookah smoking status**		**Illicit drug use**	
	**OR (95 % CI)**	***P***	**OR (95 % CI)**	***P***	**OR (95% CI)**	***P***
Univariate analysis	0.87 (0.83-0.90)	<0.001	0.90 (0.87-0.93)	<0.001	0.87 (0.83-0.92)	<0.001
Multivariate analysis	0.88 (0.84-0.91)*	<0.001	0.91 (0.88-0.94)*	<0.001	0.90 (0.85-0.95)*	<0.001

OR: odds ratio, CI: confidence interval. * Adjusted for gender, living with parents and having at least one smoker in the family based on DAGitty analysis.
